# U2 snRNP inhibition hampers pancreatic cancer cell viability by disrupting DNA repair proficiency and mitotic checkpoint integrity

**DOI:** 10.1186/s13046-026-03795-3

**Published:** 2026-07-31

**Authors:** Valentina Panzeri, Marco Pieraccioli, Veronica Ruta, Maria Vittoria Manno, Cinzia Caggiano, Duaa Hartem, Alessia Riente, Giacomo Milletti, Giuseppe Quero, Sergio Alfieri, Juan Carlos López-Gil, Ana Galván-del-Rey, Carmen Guerra, Giuseppe Maulucci, Claudio Sette

**Affiliations:** 1 Department of Neuroscience, Section of Human Anatomy, Catholic University of the Sacred Heart, Rome, 00168 Italy; 2 GSTeP Organoids Research Core Facility, Fondazione Policlinico Universitario Agostino Gemelli IRCCS, Rome, 00168 Italy; 3 Department of Basic Biotechnological Sciences, Intensive Care and Perioperative Clinics Research, Catholic University of the Sacred Heart, Rome, 00168 Italy; 4 DNA Replication and Cancer Group, Danish Cancer Institute, Copenhagen, 2100 Denmark; 5 Department of Translational Medicine and Surgery, Catholic University of the Sacred Heart, Rome, 00168 Italy; 6 Digestive Surgery Unit, Fondazione Policlinico Universitario Agostino Gemelli IRCCS, Rome, 00168 Italy; 7 Experimental Oncology group, Tumor Biology Programme, Spanish National Cancer Research Center (CNIO), Madrid, 28029 Spain; 8 Área Cáncer, Centro de Investigación Biomédica en Red (CIBERONC), ISCIII, Madrid, Spain

**Keywords:** Pancreatic cancer, SF3B1, Splicing inhibitors, Mitotic defects, Patient-derived organoids

## Abstract

**Background:**

Pancreatic ductal adenocarcinoma (PDAC) is an aggressive cancer with remarkably poor prognosis. Splicing dysregulation is emerging as a hallmark and possible therapeutic target of PDAC.

**Methods:**

We performed high-throughput RNA sequencing analyses in PANC-1 cells treated with three drugs targeting the U2 small nuclear ribonucleoprotein (U2 snRNP): Pladienolide B, Thailanstatin A and Indisulam. The efficacy of splicing inhibitors, Talazoparib, Alisertib, AZD7762 and Barisertib, alone or in combination, was assessed in PDAC cells and patient-derived organoids (PDOs) by cell viability assays. RT-PCR, western blot and cell cycle assays were performed to evaluate the expression of DNA repair and mitotic genes and the execution of mitosis. PANC-1 cells were engineered to stably co-express GFP-tagged H2b histone and RFP-tagged β-tubulin by CRISPR/Cas-9 technology and used for time-lapse confocal microscopy and immunofluorescence analyses.

**Results:**

Genes encoding for the U2 snRNP, which recognizes the 3’ splice site, are upregulated in advanced PDAC. U2 snRNP-targeting drugs impair the DNA damage response (DDR) pathway in homologous recombination-proficient PDAC cells and PDOs, thus enhancing their sensitivity to PARP inhibitors. Genes whose splicing was disrupted by all three drugs are characterized by higher expression, more exon-intron junctions, weak 5’ splice sites and atypical GC content. These features are enriched in mitotic genes (e.g. *AURKA*, *AURKB*, *CHEK1*), whose expression is strongly deregulated by splicing perturbation. Accordingly, splicing inhibition causes severe mitotic defects, leading to the accumulation of cells with misaligned condensed chromosomes on monopolar or bipolar spindles. Notably, high expression of AURKA, AURKB and CHEK1 correlates with poor prognosis in PDAC patients, whereas combined treatments with inhibitors of these mitotic kinases strongly impair the viability of PDAC cells and PDOs.

**Conclusions:**

These findings uncover gene features associated with sensitivity to splicing inhibitors in PDAC cells and suggest that the DDR and mitosis are biological processes highly sensitive to splicing perturbation.

**Supplementary Information:**

The online version contains supplementary material available at https://doi.org/10.1186/s13046-026-03795-3.

## Introduction

Pancreatic ductal adenocarcinoma (PDAC) is among the deadliest cancers, with a 5-year survival rate of ∼10%. Such dismal prognosis is related to late diagnosis, low response to chemotherapy and the lack of targeted therapies [1, 2]. The most efficacious treatment for PDAC remains surgical resection. However, more than 80% of patients present with advanced disease at diagnosis and are not eligible for surgery. The therapeutic option for unresectable patients is polychemotherapy with DNA damaging agents and taxanes, which impair genome integrity and mitotic spindle dynamics in proliferating cells [1, 2]. Nevertheless, PDAC rapidly acquires resistance to treatments and progresses to more advanced stages, for which no cure is available. Thus, development of more efficacious therapeutic options for PDAC is an urgent clinical priority [2].

Dysregulation of splicing, the process that removes the non-coding introns from the precursor messenger RNAs (pre-mRNAs), is a hallmark of cancer, which contributes to all aspects of tumorigenesis [3]. The two most frequent PDAC-associated mutations (KRAS^G12D^ and TP53^R175H/G^) were shown to synergize through a splicing program that fuels oncogenic KRAS signaling and is orchestrated by the RNA binding protein (RBP) hnRNPK [4]. Moreover, induction of the splicing factor SRSF1 during pancreatic inflammation, a well-recognized risk factor for PDAC [1, 2], accelerates KRAS^G12D^-driven tumorigenesis by promoting splice variants that support inflammation and mitogen activated protein kinase (MAPK) signaling [5]. Notably, SRSF1 expression is also induced by treatment with gemcitabine, a first-line chemotherapeutic agent for PDAC, and maintains active MAPK signaling upon genotoxic stress through splicing of the *MNKN2* gene [6]. On the other hand, expression of RBFOX2 in primary PDAC lesions was shown to restrain metastases by inducing splice variants of cytoskeletal proteins that reduce tumor cell migration and invasion [7, 8]. More recently, we have shown that a specific splicing signature clearly defines the two main cell-intrinsic molecular subtypes of PDAC [9], named classical and basal-like [10]. Expression of Quaking (QKI) in basal-like tumors, which display low response to chemotherapy and worse clinical course, promotes the expression of splice variants associated with advanced disease and poor prognosis [9]. Collectively, these studies suggest that splicing dysregulation is a main determinant of pancreatic tumorigenesis [11].

A targetable node in splicing regulation is the U2 small nuclear ribonucleoprotein (U2 snRNP), the spliceosome particle that recognizes the splice site at the 3’ end of introns [12]. Several natural anticancer compounds were demonstrated to bind and inhibit splicing factor 3 b (SF3B) [13], the component of the U2 snRNP involved in recognition of the branchpoint sequence upstream of the 3’ splice site [12]. Furthermore, the *SF3B1* gene is frequently mutated in hematological malignancies and, albeit at much lower frequency, in several solid tumors [13–16], including PDAC [17]. Mutant SF3B1 impacts on the glycolytic metabolism of pancreatic cells and enhances tumorigenesis triggered by oncogenic mutations [18, 19]. At the same time, up-regulation of *SF3B1* expression in the PDAC cases that do not harbor mutations in this gene correlates with disease progression and with widespread splicing changes in genes related to tumorigenesis [20]. Notably, cancer cells overexpressing SF3B1, or harboring mutations in this gene, were found to be particularly sensitive to inhibitors that target the U2 snRNP [11, 14, 15, 21]. Herein, by interrogating single-cell transcriptomic data we found that the expression of other components of the U2 snRNP, such as SF3A1, SF3B4 and the U2 accessory factor of 65 kDa (U2AF65 or U2AF2) are upregulated in advanced and metastatic PDAC, pointing to a pro-oncogenic role for U2 snRNP in this cancer.

Splicing inhibitors were shown to generate cancer cell-specific vulnerabilities to agents that are currently in the clinic, such as DNA damaging agents, taxanes and poly-ADP-ribose polymerase inhibitors (PARPi) [22–27]. Nevertheless, whether this approach is valuable in PDAC, which is particularly resistant to conventional chemotherapy, has not been directly investigated. Herein, we have tested the effects of splicing inhibition on PDAC cell fitness by employing two drugs targeting SF3B1, Pladienolide B (PdB) and Thailanstatin A (ThA) [28, 29], and indisulam, a molecular glue that targets RBM39 [30], an U2AF65-like RBP [14, 15]. Transcriptome and functional analyses in PDAC cell lines uncovered gene features and biological processes that are highly susceptible to splicing perturbation. Moreover, studies in PDAC cells and patient-derived organoids (PDOs) revealed that genetic and pharmacologic inhibition of U2 snRNP factors strongly impacts on the completion of the cell cycle, by disrupting the expression of proteins involved DNA repair and mitosis, and uncovered splicing-sensitive actionable targets that trigger synthetic lethality in PDAC cells.

## Materials and methods

### Cell culture, transfection and treatments

PANC1 cells were cultured in RPMI 1640 medium (Euroclone); AsPC-1, MiaPaCa-2 and CFPAC cells were grown in D5796 Dulbecco’s modified Eagle’s medium (DMEM; Sigma Aldrich); HPAF-II were cultured in Eagle’s minimum essential medium medium (EMEM; Sigma Aldrich) with sodium pyruvate. All media were supplemented with 10% fetal bovine serum (FBS; Gibco), non-essential amino acids (Thermo Fischer), penicillin and streptomycin and gentamicin (Thermo Fischer). Cells were grown in a 37 °C humidified atmosphere with 5% CO_2_. For RNA interference (RNAi), cells were transfected with the indicated siRNAs (siAURKA GCAAUUUCCUUGUCAGAAU[dT][dT]; siAURKB CGCGGCACUUCACAAUUGA[dT][dT]; siCHEK1 GGGAUAUUAAACCAGAAAA[dT][dT]; siSF3B1 GUGUAGAACUGGUCAUAGA[dT][dT]); siRBM39 GCAGCAAUGGCAAACAAUU[dT][dT] using Lipofectamine RNAiMAX (Thermo Fisher Scientific). Cells were treated with Pladienolide B (Santa Cruz; sc-391691), Thailanstatin A (MedChemExpress; HY-129589), Indisulam (MedChemExpress; HY-13650), CHEK1 inhibitor (AZD7762), Alisertib (Selleckem; S1133), Barisertib (Selleckem; S1147), Talazoparib (Pfizer; CAS number 1373431-65-2) at the indicated doses, or dimethylsulphoxide (DMSO) as a control.

### CRISPR/Cas9-based engineering of PANC-1 cells

For the generation of PANC-1 cells stably expressing H2B-GFP and Tubulin-RFP, we employed the following plasmids: H2BC11-GFP: pX330-PITCh-H2BC11 Cas9 plasmid (Addgene # 207755) and qTAG-C-moxGFP-Puro-H2BC11-MMEJ repair plasmid (Addgene # 207760); TUBULIN-RED: pX330-PITCh-TUBA1B Cas9 plasmid (Addgene # 207763) and qTAG-N-Blast-sTagRFP-TUBA1B repair plasmid (Addgene # 207768). Plasmids were transfected with Lipofectamine 3000 with 1:1 ratio for a total of 3ug. Media replacement occurred the next day and an editing period of 72 h post-transfection was allowed prior to selection enrichment (Blasticidin or Puromycin). The selected clones were amplified and checked at fluorescence microscope [31].

### Patient-derived organoids

Organoids were derived from tumor biopsies from patients undergoing surgery as curative treatment at the Fondazione Policlinico Universitario A. Gemelli IRCCS (FPG), Rome, Italy. The protocol (ID: 4121) was approved by the Institutional Review Board and conducted in accordance with the Helsinki Declaration. All patients enrolled gave their written informed consent for participation to the study. As previously described [9], tumor tissues were placed in 60 mm Petri dishes containing AdDF+++ culture medium (Advanced DMEM/F12 containing 1x Glutamax, 10 mM HEPES and antibiotics). Part of the tissue was fixed in formalin for histopathological and immunohistochemistry analysis; part was stored at -80 °C for DNA/RNA isolation. The remaining part was minced by surgical blades into small fragments and digested in 10 mL AdDF+++ supplemented with 5 µM RHO/ROCK pathway inhibitor (Y-27632, Tocris) containing 2 mg/mL Collagenase II (Thermo Fisher Scientific), Dispase and DNAse I (Sigma-Aldrich) on an orbital shaker at 37 °C for 1 h. The cell suspension was then applied to a MACS SmartStrainer (70 mm), placed on a 50 mL tube and washed with 10 mL of AdDF^+ + +^ culture medium and centrifugated at 290 *g* at 4 °C. The pellet was incubated with 1 mL red blood cell lysis buffer for 5 min at room temperature to eliminate erythrocytes, followed by washing with culture medium and pelleting at 4 °C for 290 g. Cells were embedded in undiluted (100%) Cultrex growth factor reduced BME type 2 (Bio-Techne) on ice and 40 µL drops of BME cell suspension were allowed to solidify to a pre-warmed 24 well suspension culture plates (Greiner). The plate was placed at 37 °C for 30 min to allow the Matrigel to polymerize before being overlaid with 500 µL of a growth factor cocktail medium [AdDF+ + +culture medium supplemented with A83-01 (0.5 µM), B27 (1X GIBCO), Primocin (1 mg/mL, InvivoGen), Y-27632 (9 µM Tocris), Wnt3a-conditioned medium (50% v/v), RSPO1-conditioned medium (10% v/v), Nicotinamide (10 mM, Sigma-Aldrich), Gastrin (10 nM, Biogems), fibroblast growth factor 10 (FGF10, 100 ng/mL, Peprotech), N-acetyl-L-cysteine (1 mM, Sigma-Aldrich) and Noggin (0.1 mg/mL, Peprotech)] and incubated at 37 °C in humidified air containing 5% CO_2_ until organoids were formed. Medium was changed every 3–4 days and organoids were passaged every 1–2 weeks at suitable ratio (1:2 to 1:3). Somatic mutations of original tumor tissue PDOs were assessed by TruSight Oncology 500 (Illumina). Viability of PDOs was assessed as previously described [32]. Briefly, after the dissociation by Triple Express, organoid fragments were resuspended in 2% BME/growth medium and seeded in 100 µl volume on BME pre-coated 96-well plates. After 24 h, samples were treated with the indicated drugs for 5 days and viability was determined by CellTiter-Glo^®^ Luminescent Cell Viability Assay (Promega) using a microplate reader (Spark, Tecan). PDOs were classified as small, medium, or large based on their diameter measured manually drawing lines with ImageJ software. To calculate the organoid diameter range, we subtracted the diameter of the smallest organoid from the diameter of the largest organoid and divided this value by 3. The obtained value (n) was used to determine the diameter range for small (1n), medium (2n), and large organoids (3n) [33].

### RNA sequencing and bioinformatics analysis

Total RNA was extracted and DNase treated using the RNAeasy mini kit (QIAGEN) from PANC-1 cells treated for 6 h for PdB (1 nM), 8 h for ThA (200 nM) and 12 h for Indisulam (3 µM) or DMSO as control. PolyA plus RNA-seq libraries were constructed using the Universal Plus™ mRNA-Seq library preparation kit with NuQuant^®^ (Tecan) and sequenced using a 150 bp paired-end format on an Illumina NovaSeq 6000 and analysis was performed as previously described [9, 25, 26]. Splicing analyses were performed considering only exon and intron reads (“EXON” analysis) and flanking exon-exon junction reads to detect new potential alternative events and known patterns (“PATTERN” analysis) using the Human CAST DB v4 splicing patterns annotation. Results were considered statistically significant for p-values ≤ 0.05, fold-changes ≥ 1.5 and absolute delta PSI (Percent Spliced Index) ≥ 10 for “PATTERN” analysis and p values ≤ 0.01 and fold-changes ≥ 2.0 for “EXON” analysis. Analysis of length was calculated by considering the start and end coordinates for each gene, exon or intron analyzed and expressed as log2. Analysis of 5’ splice-sites strengths were scored using MaxEntScan tool [34]. Analysis of GC content for each gene, exon or intron sequence was obtained by weighing the GC count with their length. Analysis of Branch Point Score from acceptor splice-site were evaluated using SVM-BPfinder [35].

The analysis of AURKA, AURKB, and CHEK1 transcript expression in the Basal-like and Classical-like subtypes was performed using the Clinical Proteomic Tumor Analysis Consortium (CPTAC) dataset [36].

Single cell sequencing data from previously published dataset [37] were downloaded from the Gene Expression Omnibus under the accession code GSE205013 (PDAC patients *n* = 27). Data were pre-processed as previously described [9, 37]. For analysis, only cells annotated as “Epithelial cluster” were used and cells of each patient were classified to the classical, intermediate or basal-like subtypes and in “NoMetastatic” or “Metastatic” group as described by the authors. Dot plot and violin plots were generated using the R package Seurat version 4.3.0.

### Reverse transcription–polymerase chain reaction (RT–PCR)

Total RNA was extracted using Trizol reagent (Life Technologies-Thermo Fisher Scientific) or with the ‘Total RNA Mini Kit’ (Geneaid). After digestion with RNase-free DNase (Life Technologies-Thermo Fisher Scientific), RNA was resuspended in RNase-free water (Sigma Aldrich) and reverse transcribed (1 µg) using M-MLV reverse transcriptase (Promega) and oligo(dT) primers (Roche). 20 ng of cDNA was used as template for both conventional PCR analysis (GoTaq, Promega). PCR primers are listed in Additional file 1.

### Protein extraction and western blot

Cell pellets were resuspended in RIPA buffer [50 mM Tris–HCl, 1% NP-40, 150 mM NaCl, 0.5% Na-deoxycholate, 2 mM EDTA and 0.1% sodium dodecylsulphate (SDS)] supplemented with 2 mM Na-orthovanadate, 0.5 mM sodium fluoride and Protease Inhibitor Cocktail (Sigma Aldrich), 1 mM dithiothreitol (DTT) or 1 mM TCEP [Tris(2-carboxyethyl)phosphine]. After 10 min incubation on ice, extracts were sonicated at maximum intensity for 5 s, centrifuged for 10 min at 15,000 g (4 °C) and supernatant fractions were resuspended in SDS–polyacrylamide gel electrophoresis (PAGE) sample buffer and boiled for 10 min. The lysates were separated by SDS–PAGE, transferred to polyvinylidene fluoride (PVDF) membranes and incubated with primary antibodies (overnight at 4 °C) for the following proteins: BRCA1 (Cell Signaling, BK9010S), BRCA2 (Millipore; OP95), CHEK1 (Cell Signaling; BK2360S), AURKA (Cell Signaling; 14475), AURKB (Cell Signaling; BK3094S), Cleaved caspase-3 (Cell Signaling, 9661), PARP1 (Cell Signaling, 9542), γH2AX (Cell Signaling, BK9718S), SF3B1 (Bethyl, A300-996 A), RBM39 (Atlas, HPA001591), HSP90 (Santa Cruz, sc-13119). Secondary anti-mouse or anti-rabbit IgGs conjugated to horseradish peroxidase (Amersham, UK) were incubated for 1 h at room temperature (1:5000). Immunostained bands were detected by the chemiluminescence method.

### Cell viability and cell cycle assays

Cells were seeded in a 96-well plate and imaged at ×10 magnification using the IncuCyte SX5 Live-content imaging system (Essen Bioscience) at 37 °C with 5% CO_2_. For cell viability assays, the images were acquired every 6 h for 4 days (four images/well) and analysed using the Cell-by-Cell analysis software to detect and quantify live cells. For cell cycle analysis, cells were labelled with 10 μm Bromodeoxyuridine (BrdU) for 30 min and then fixed in 70% ethanol–phosphate-buffered saline (EtOH-PBS) and stored at − 20 °C overnight. DNA content was evaluated by flow cytometry using 7-aminoactinomycin D (7-AAD; 2.5 µg/ml, Biotium) and anti-BrdU antobody (0.125 µg × sample) in the presence of RNase A (1 µg/ml). A total of 20 000 events were counted with a CytoFlex flow cytometer (Beckman) and analysed using CytExpert software.

### Immunohistochemical analysis

PDOs were included in Histogel (Epredia, HG-4000-012) and then paraffin embedded. PDOs stained with haematoxylin and eosin (H&E) and immunohistochemistry staining was performed with primary antibodies: KI-67 (Abcam, ab16667), KRT-18(Sigma, hpa001605), MUC-13 (Invitrogen, PA5-104505). The signal is detected by UltraView Universal DAB Detection Kit on the ULTRA instrument (Ventana) BenchMark.

### Immunofluorescence analyses

For immunofluorescence analysis, PANC-1 cells were fixed with 4% paraformaldehyde and permeabilized with 0.1% Triton X-100. After 1 h at room temperature (RT) in blocking buffer (PBS, 5% BSA) it is incubated for 90 min with gH2AX antibody (1:500) in PSB 1% BSA. After rinsing, secondary anti-rabbit Alexa Fluor 594 (1:500; Thermo Fisher Scientific) antibodies diluted in PBS supplemented with 1% BSA were incubated for 1 h at RT. Nuclei were counterstained with Hoechst 33,342. For quantitative analysis of cleaved caspase 3 staining, we employed an automated multichannel wide-field microscopy for high-content imaging and quantitative image-based cytometry (QIBC). The analysis was performed using the CellDiscoverer 7 (Zeiss) with the Plan-Apochromat 5x Objective, set with the internal 2× magnification changer to yield a total 10× magnification (Numerical Aperture NA = 0.35). Cells were labelled with Caspase-3 Dyes (Sartorius; 4440) and segmented with ArivisVision4D software, version 4.1.1. using a customized pipeline implemented with the cellpose algorithm.

### Live-image acquisition

Live-cell confocal imaging was performed using a Nikon A1-MP microscope equipped with a 40× dry objective, delivering 80-fs pulses at an 80 MHz repetition rate. Time-lapse acquisitions were carried out over a 72-hour period under controlled environmental conditions (37 °C, 5% CO₂) maintained by an on-stage incubation system (OKOLAB S.r.l., Pozzuoli, Italy). To ensure continuous focus stability throughout the time-lapse imaging, the Nikon Perfect Focus System (PFS) was employed. Two fluorescence detection channels were configured: Channel 1 (Ch1) with excitation at 402.6 nm and emission collected through a 450/50 nm bandpass filter, and Channel 2 (Ch2) with excitation at 488.3 nm and emission detected through a 525/50 nm bandpass filter. Fluorescently labeled tubulin was monitored in Ch1, while GFP-tagged histone H2 was visualized in Ch2. Image sequences were acquired for both control and treated samples. Acquisition loops were programmed at 13-minute intervals, and all images were recorded at a spatial resolution of 2048 × 2048 pixels.

### IQGAP3 minigene assay

The IQGAP3 genomic region from exon 33 to exon 35 was amplified using Phusion Hot Start High-Fidelity DNA polymerase (Thermo Fisher Scientific) and cloned into the XhoI/MluI restriction sites of pCI vector (Promega). The minigene mutated for the 5’ splice site of exon 34 was generated by the megaprimer strategy. All primers are listed in Additional file 1. PANC-1 cells were transfected using Lipofectamine 2000 (Thermo Fisher Scientific) with 250 ng/well of wild type or mutated minigene in a 6-well plate at 70% cell confluency and treated after 24 h with the indicated doses of PdB for additional 6 h.

### Statistical analysis

Statistical analyses were conducted using GraphPad Prism 9.0.2 or R packages ggpubr 0.4.0, DESeq2 1.32.0 and Aspli 2.2.0. The number of replicates and specific statistical tests are detailed in each figure legend. A p-value ≤ 0.05 was considered significant, statistical significance was defined as *P ≤ 0.05, **P ≤ 0.01, ***P ≤ 0.001.

Synergy between Drug A and Drug B was evaluated using the Bliss independence model. Values were expressed as the difference between the expected and the observed effect, the cutoff of 0.10 was used as threshold for synergy between drugs, while values between 0 and 0.1 indicated additivity between the drugs [38].

## Results

### Splicing inhibition impairs the DNA damage response in PDAC cells

Splicing is emerging as a potential therapeutic target in PDAC [11, 39], with upregulation of SF3B1 being associated with disease progression [20]. By querying single-cell transcriptomic data [37], we observed that most genes encoding for U2 snRNP proteins (8 out of 10) are significantly upregulated in metastatic PDAC with respect to primary, non-metastatic tumor cells (Fig. S1A, B). A similar up-regulation was also observed for *SF1*, encoding for the protein that recognizes the branch-point [12], and *U2AF2*, encoding for U2AF65 (Fig. S1A, B). U2 snRNP genes were also upregulated in the more aggressive intermediate and basal-like cells with respect to classical PDAC cells (Fig. S1C). Moreover, a U2 snRNP signature comprising the 8 genes upregulated in metastatic PDAC predicted poor prognosis in patients (Fig. S1D). Together, these data suggest an oncogenic function for U2 snRNP in PDAC. To directly test this hypothesis, we performed dose-response studies with U2 snRNP-targeting drugs in three cell lines that are representative of medium (AsPC-1) and high (PANC-1 and MiaPaCa-2) grade tumors [40, 41]. All cell lines displayed a half-maximum inhibitory concentration (IC_50_) in the low nanomolar range for the SF3B1 inhibitors PdB and ThA, and in the low micromolar range for indisulam (Fig. S1E). Moreover, splicing inhibitors induced cell death at suboptimal doses, as shown by cleavage of PARP-1 and caspase 3 proteins (Fig. S1F).

**Fig. 1:**
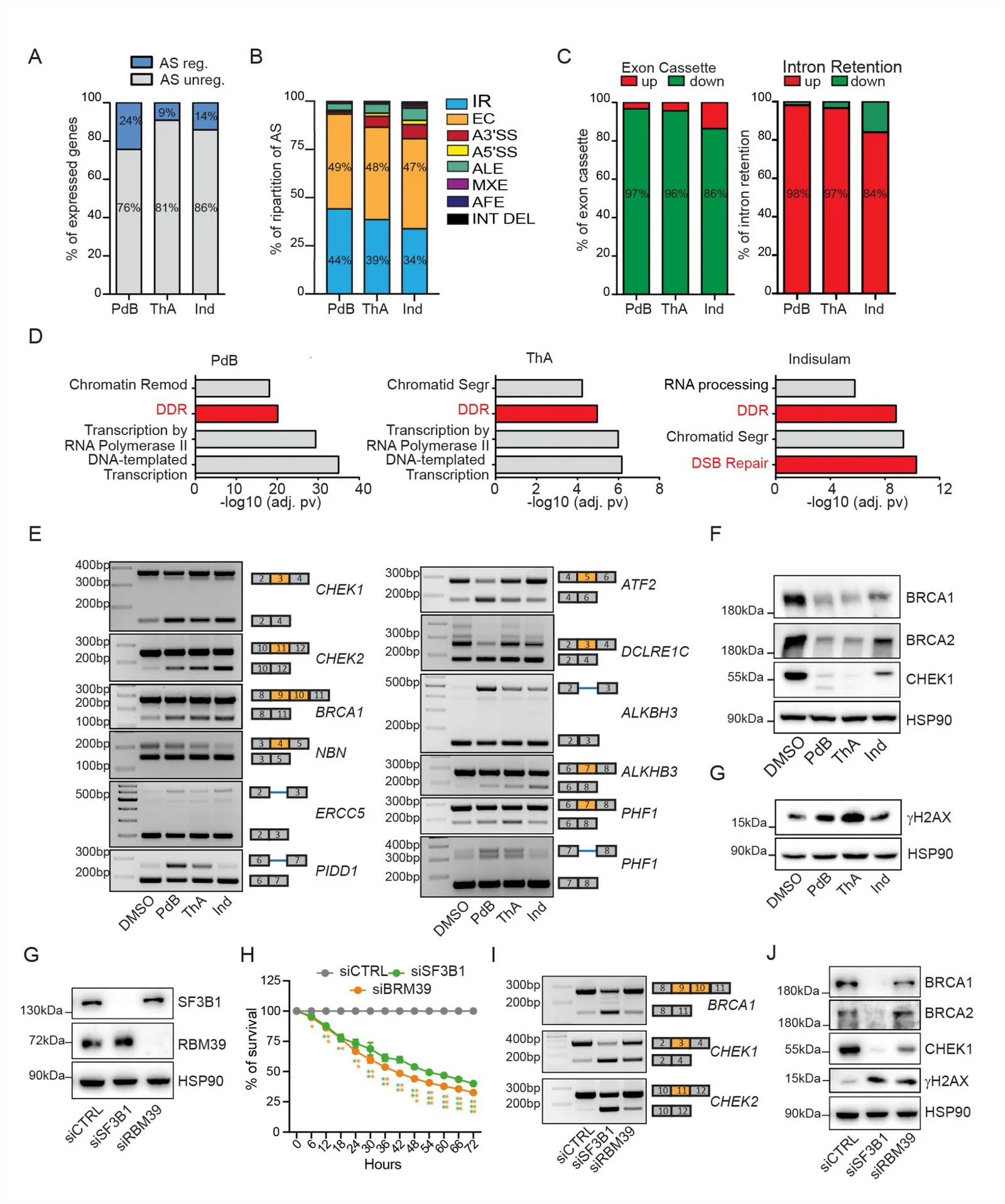
Splicing inhibitors impair the DNA damage response in PDAC cells. **A** Bar graphs representing the percentage of expressed genes regulated by alternative splicing after treatment with 3.3nM Pladienolide **B** (PdB) for 6 h, 500nM Thailanstatin A (ThA) for 8 h and 3.3uM Indisulam (Ind) for 12h. **B** Percentage of the indicated splicing patterns regulated by each splicing inhibitor. IR, intron retention; EC, exon cassette; A3’SS, alternative 3’ splice site; A5’SS, alternative 5’ splice site; ALE, alternative last exon; MXE, Mutually Exclusive Exon; AFE, alternative first exon; INT DEL, internal exon deletion. **C** Bar graph showing percentages of up- or down-regulated r exon cassette and intron retention events in PANC-1 cells treated as indicated. **D** Bar graph showing the GO terms relative to biological processes enriched within the splicing regulated genes by the indicated drugs. **E** RT-PCR analysis for representative splicing events affected by PdB, ThA and Ind in PANC-1 cells. **F** Representative Western Blot analysis for BRCA1, BRCA2, CHEK1 and HSP90 protein levels in PANC-1 cells treated with PdB (15 h), ThA (15 h) and Ind (24 h). **G** Representative Western Blot analysis for γH2AX and HSP90 protein levels in PANC-1 cells treated for 72 h with splicing inhibitors. **G** Western Blot analysis for SF3B1, RBM39 and HSP90 protein levels in PANC-1 cells transfected with the indicated small interfering RNAs (siRNAs) (48 h). **H** Cell proliferation analysis of PANC-1 cells transfected with the indicated siRNAs. Data represent the mean *±* SD of three independent experiments. Statistical analyses were performed by two-way ANOVA; *P ≤ 0.05, **P ≤ 0.01, ***P ≤ 0.001. Values are reported in green when referring to siSF3B1 and in orange when referring to siRBM39 transfected cells. **I** RT-PCR analysis for representative splicing events affected by transfection of siSF3B1 and siRBM39 in PANC-1 cells. **J** Western Blot analysis for BRCA1, BRCA2, CHEK1, γH2AX and HSP90 protein levels in PANC-1 cells transfected with siSF3B1 and siRBM39 (48 h)

To identify pathways underlying the pronounced toxicity of splicing inhibitors in PDAC cells, we performed high-throughput RNA sequencing (RNA-seq) analyses in PANC-1 cells treated for short time with PdB (3.3 nM; 6 h), ThA (500 nM; 8 h) and indisulam (3.3 µM; 12 h). All three drugs elicited a strong impact on the PDAC transcriptome, with 24% of the expressed genes being altered at splicing level by PdB, followed by indisulam (14%) and ThA (9%; Fig. 1A). Splicing inhibitors prevalently induced skipping of exon cassette (EC) and intron retention (IR; Fig. 1B, C). Gene ontology (GO) analysis highlighted RNA transcription and DNA damage response (DDR) as the biological processes mostly affected by these drugs (Fig. 1D). We focused on the DDR pathway, as it is directly involved in the response to the chemotherapeutic drugs in use for PDAC [1, 2]. RT-PCR analyses confirmed that splicing of DDR genes is altered by at least one of the drugs (Fig. 1E, Fig. S1G), including those encoding for proteins that signal the DNA lesion, such as CHEK1 and CHEK2, or promote the assembly of the repair machinery, such as BRCA1 (Fig. 1E). These splicing changes exert a functional impact, as they were accompanied by a pronounced reduction of the BRCA1, BRCA2 and CHEK1 proteins (Fig. 1F, Fig. S1H) and accumulation of DNA double strand breaks (DSBs), as shown by phosphorylation of H2AX (gH2AX; Fig. 1G, Fig. S1I) [42]. Importantly, knockdown of SF3B1 or RBM39 recapitulated these effects on the DDR pathway (Fig. 1G-J), thus confirming the on-target effects of the three splicing inhibitors.

### Splicing inhibition sensitizes PDAC cells and organoids to PARP inhibitors

We previously showed that a transient pulse with splicing-targeting drugs exerts reversible splicing inhibition, while persistently hampering the fitness of cancer cells with high genomic instability [26]. To test this possibility in the context of PDAC, we exposed PANC-1 cells to PdB (3nM) for 16 h and then released them from the drug for up to 48 h. Under these conditions, splicing of DDR genes was rescued, whereas the expression of DDR proteins was persistently repressed (Fig. S2A, B), suggesting that transient treatment with splicing inhibitors could generate a status of homologous recombination deficiency (HRD) in otherwise proficient cells.

PDAC patients harboring inactivating mutations in *BRCA1/2* genes can benefit of PARP inhibitor (PARPi)-based therapies [43]. Thus, we asked whether splicing inhibitors could sensitize PDAC cells to the clinically approved PARPi Talazoparib [44]. Notably, pre-treatment (24 h) of PANC-1 cells with suboptimal doses of all three splicing inhibitors enhanced their response to Talazoparib (Fig. S2C, D). Since continuous combined treatment exerted similar effects (Fig. S2E), these data indicate that a short pulse of splicing inhibition is sufficient to elicit long-lasting repression of the DDR in PDAC cells.

**Fig. 2:**
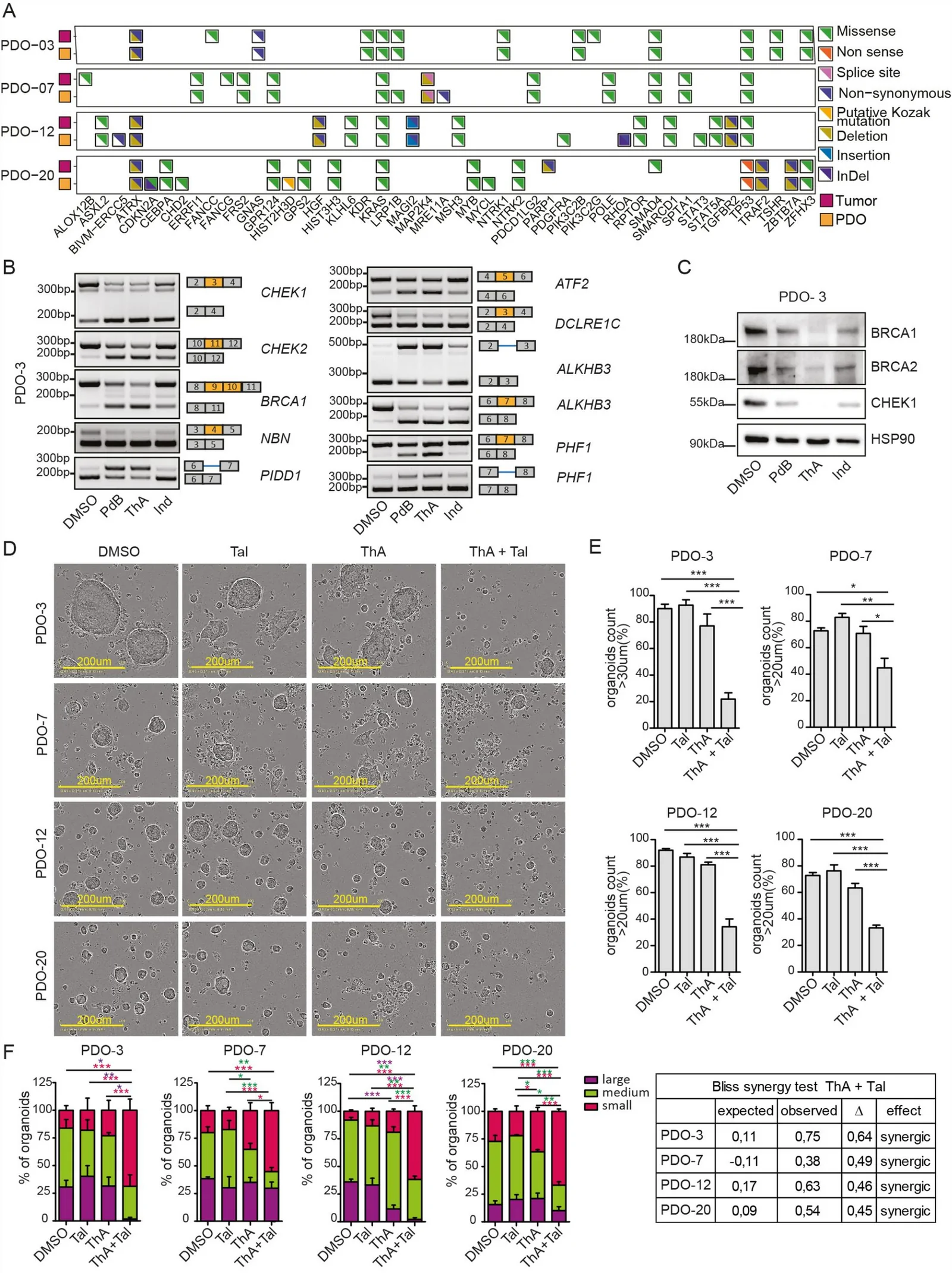
Splicing inhibitors sensitize PDAC patient-derived organoids to PARP. **A** Somatic mutations and amplifications/deletions found in the indicated PDOs. For each patient’s sample, tumor/organoid pairs are displayed and indicated by color coding (pink, tumors; orange, organoids). **B** RT-PCR analyses for representative splicing events affected by of PdB, ThA and Indisulam treatment (24 h) in PDO-3. **C** Representative Western Blot analysis for BRCA1, BRCA2, CHEK1 and HSP90 protein levels in PDO-3 treated for 48 h with PdB, ThA and Indisulam. **D** Bright-field images of PDO cultures treated for 5 days with 200nM of Talazoparib (Tal), 300nM (PDO-3-7-12) or 400nM (PDO-20) ThA alone or in combination (combo). Scale bar, 200 μm. **E** Percentages of organoids that have a size greater than 30 μm (PDO-3) or 20 μm (PDOs 7-12-20). Synergism between drugs was evaluated by the Bliss independence model, setting the Δ between expected and observed > 0.1 as threshold. **F** Percentage of large, medium, and small PDOs for the conditions described in **D**. Statistical analyses are reported in purple, green and pink when referring to large, medium and small organoids respectively. Statistical analyses were performed one-way ANOVA (**E**) or two-way ANOVA (**F**); *P ≤ 0.05, **P ≤ 0.01, ***P ≤ 0.001

To test whether splicing inhibition efficiently impaired the DDR also in PDAC models that are more representative of individual patient’s tumors, we developed four PDO lines from surgically resected tumor biopsies (Fig. S3A). Analysis of diagnostic markers of PDAC confirmed the neoplastic origin of these PDOs (Fig. S3B). Moreover, genomic analysis showed that the PDOs captured the mutational spectrum of the corresponding original tumor, including hotspot mutations in the *KRAS* and *TP53* genes (Fig. 2A), while they were wild type for *BRCA1* and *BRCA2*. Treatment of PDAC PDOs with splicing inhibitors recapitulated the splicing changes in DDR genes identified in PDAC cell lines (Fig. 2B, Fig. S3C). Furthermore, all three drugs caused a reduction in the expression of BRCA1, BRCA2 and CHEK1 proteins (Fig. 2C; Fig. S3D). We then selected the SF3B1 inhibitors for further studies, as they showed stronger effects on the reduction of DDR proteins in these PDOs. Combined treatment with suboptimal doses of Talazoparib and ThA strongly reduced the number of PDOs with respect to each drug used as single agent (Fig. 2D, E). Moreover, the PDOs surviving to the combined treatment were significantly smaller in size (Fig. 2F). Similar results were obtained by combined treatment with PdB (Fig. S4A-C). Importantly, the CHEK1 inhibitor AZD7762 (CHEK1i) also enhanced the efficacy of Talazoparib (Fig. S4D, E), suggesting that aberrant splicing and function of CHEK1 is involved in the synergy between splicing-targeting drugs and PARPi. Together, these findings indicate that splicing inhibition negatively impacts on the DDR pathway in HR proficient (HRP) PDAC models and represents an efficacious strategy to sensitize these tumors to treatment with PARPi.

### U2 snRNP inhibitors preferentially dysregulate highly expressed genes characterized by small exons and introns and weak 5’ splice sites

**Fig. 3:**
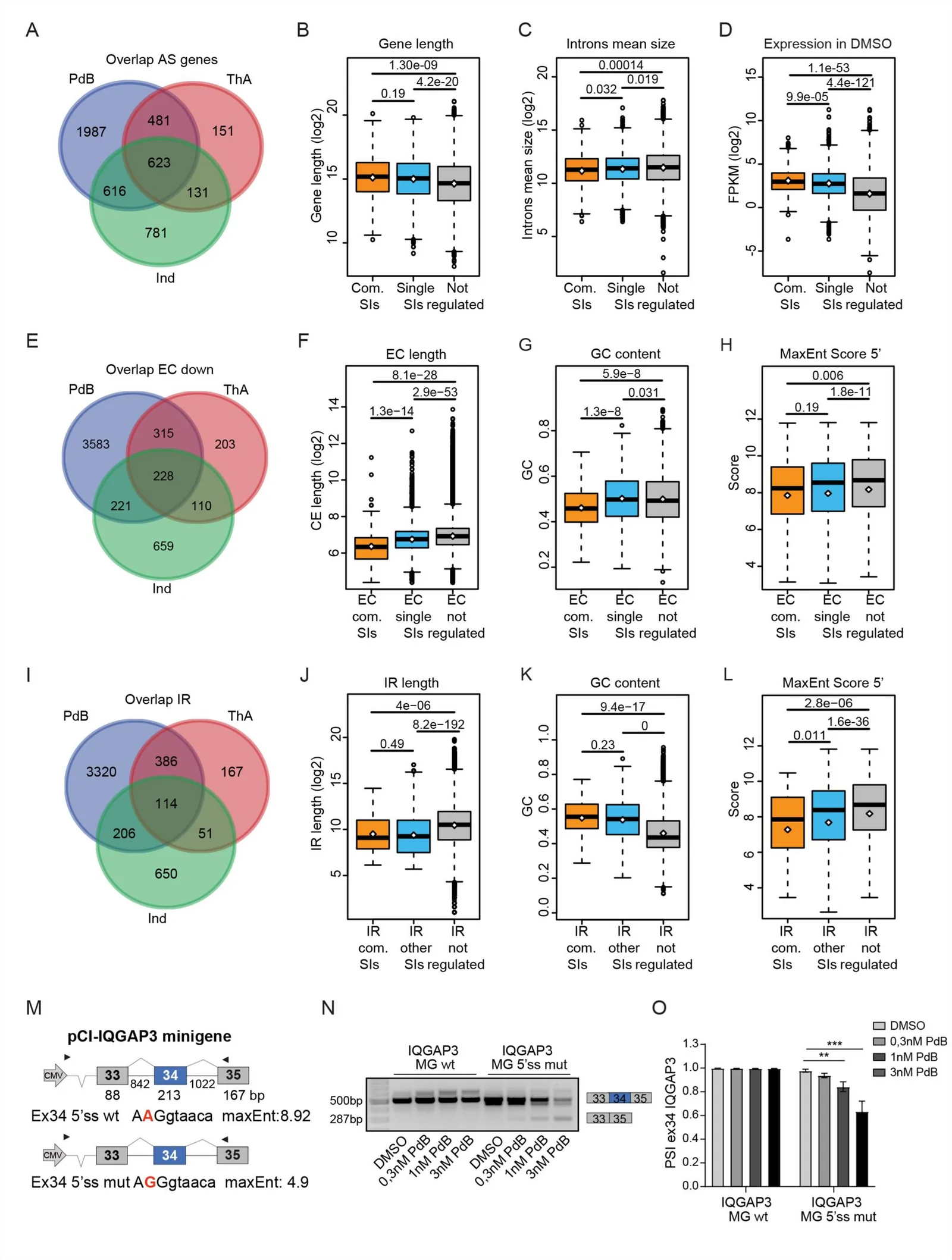
Structural features of genes regulated by U2 snRNP inhibitors. **A** Venn diagram showing the overlap between genes regulated by PdB, ThA and Indisulam. **B**-**D** Box plots representing comparison of gene length (**B**), intron mean size (**C**) and FPKM (**D**) between the 623 common splicing inhibitor (SI) regulated genes, 2987 genes regulated only by one of the splicing targeting drugs (genes other SI) and 9062 genes not regulated by any of them. **E** Venn diagram showing the overlap between exon cassette (EC) regulated in PdB, ThA and Indisulam. **F-H** Box plots representing comparison of EC length (**F**), GC content (**G**) and MaxEnt score 5’ss (**H**) between 228 EC common SI regulated, 4445 EC regulated only by one of the drugs (other SI) and 122043 EC not regulated by any of them. **I** Venn diagram showing the overlap between intron retention (IR) regulated in PdB, ThA and Indisulam. **J-L** Box plots representing comparison of IR length (**J**), GC content (**K**) and MaxEnt score 5’ss (**L**) between 228 IR common SI regulated, 4445 IR regulated only by one of the drugs (other SI) and 122043 IR not regulated by any of them. **M** Schematic representation of the construction of the minigene for IQGAP3, with the A > G mutation at the 5’ss. **N-O)** Representative RT-PCR analysis (**N**) and densitometric analysis (**O**) for IGAP3 splicing from the wt and 5’ss mut MG after 6 h of treatment with PdB at the indicated doses in PANC-1 cells. *P*-values were calculated with Wilcoxon test and p. adjusted with the Benjamini-Hochberg (BH) adjustment (panels **B**-**D**, **F**-**H**, **J**-**L**) and two-way ANOVA (panel **O**); **P ≤ 0.01, ***P ≤ 0.001

Overlap of the three RNA-seq datasets identified a core set of 623 genes whose splicing was affected by all splicing-targeting drugs (Fig. 3A; Additional file 2). To search for structural and functional features underlying the high susceptibility of the genes regulated by all three splicing inhibitors (common SI), we compared them with genes regulated only by one of the drugs (single SI) or not regulated by any of them.

While none of the three groups displayed significant differences in GC content (Fig. S5A), common SI genes were characterized by an increased length (Fig. 3B). This feature was also present in single SI genes, albeit they trended to be enhanced in the commonly regulated genes. Moreover, common SI genes were characterized by a significant reduction of mean intron length and higher expression levels with respect to the other two groups (Fig. 3C, D). Since EC skipping and IR were the most affected splicing patterns (Fig. 1C), we also evaluated the structural features associated with these splicing events. The common skipped ECs were significantly shorter and with lower GC content with respect to exons regulated by only one drug or not regulated by any of them (Fig. 3E-G). GC content of their flanking introns was also significantly reduced (Fig. S5B). Moreover, while there were no differences in 3’ ss strength, they were characterized by significantly weaker 5’ ss and branchpoint with respect to non-regulated ECs. (Fig. 3H, Fig. S5C). Common retained introns were also smaller than non-regulated ones, but they displayed a higher GC content (Fig. 3I-K). Furthermore, they were also characterized by a significantly weaker 5’ ss with respect to both other groups of introns (Fig. 3L).

To test whether these features could be directly involved in the susceptibility to splicing inhibitors, we focused on the 5’ splice site strength. We constructed a minigene comprising the genomic region between exon 33 and exon 35 of IQGAP3, including the two intervening introns (Fig. 3M). This gene was chosen because exon 34 has a strong 5’ splice site (maxEnt = 8.92) and its splicing was not regulated by PdB treatment. RT-PCR analyses using a forward primer specific for minigene-encoded transcripts and a reverse primer in exon 35 confirmed that exon 34 is completely included in transfected PANC-1 cells, while PdB treatment does not cause its skipping. However, when the 5’ splice site was mutated by only one nucleotide (A > G) to reduce its strength (maxEnt = 4.9) the transcript acquired sensitivity to PdB and exon 34 was skipped in a dose-dependent fashion (Fig. 3N, O). Together, these data suggest that high expression of long genes renders their small exons or introns with a weak 5’ ss particularly susceptible to drugs that impair the recognition of the 3’ss. Altered GC content and reduced branchpoint strength may further contribute to the enhanced dependency of these genes on a functional U2snRNP, likely due to the high number of poorly defined exon-intron junctions that need to be efficiently spliced to ensure their proper processing.

### Mitotic checkpoint genes are highly susceptible to splicing inhibition

**Fig. 4:**
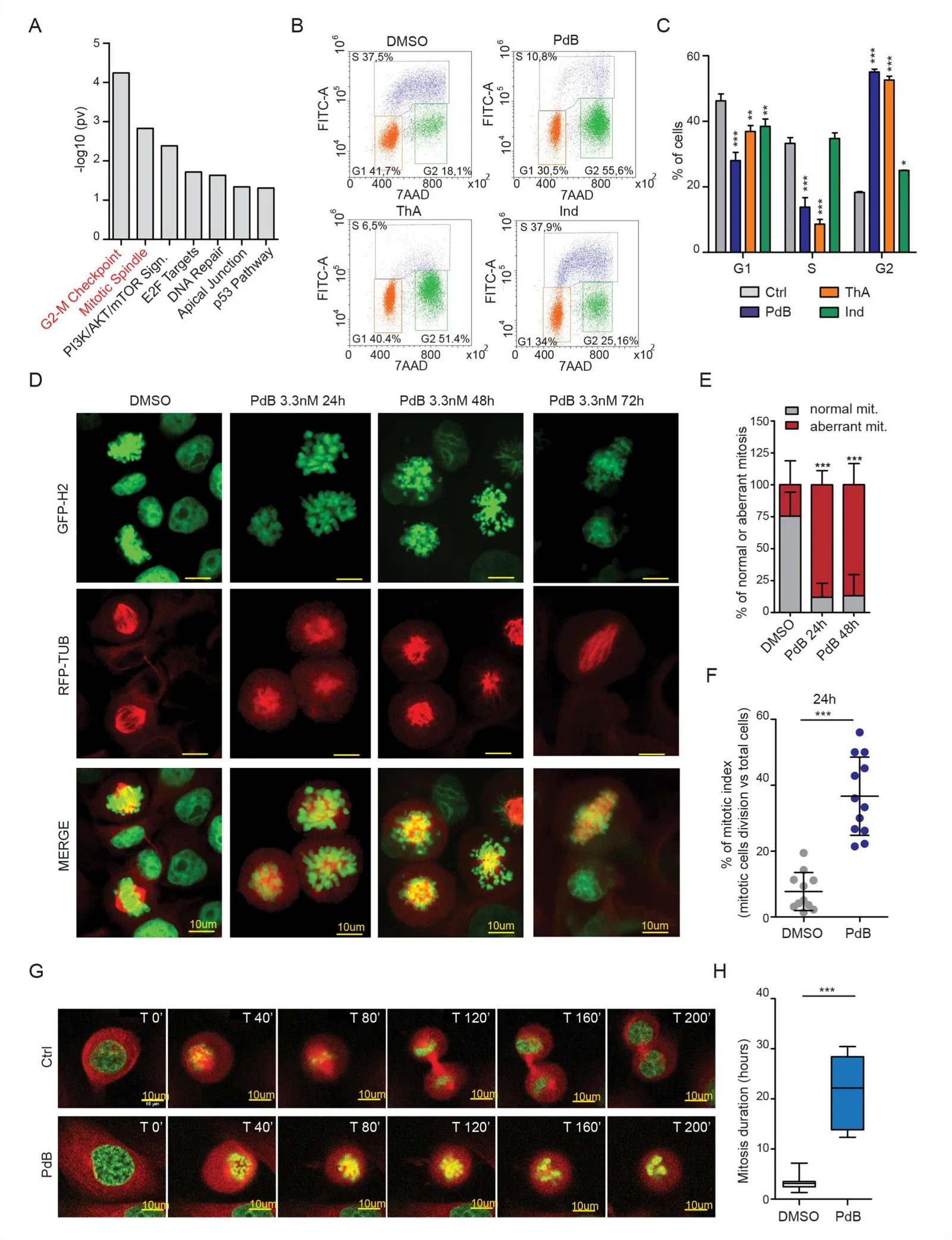
Splicing inhibitors disrupt the execution of mitosis. **A** Terms of The Molecular Signatures Database enriched in the 623 genes whose splicing is disrupted by short-term treatment with all three splicing-targeting drugs. **B** FACS analysis showing DNA content (7AAD) and bromodeoxyuridine (BrdU) incorporation of PANC-1 cells treated for 48 h with 3.3nM of PdB, 500nM of ThA and 3.3µM of Indisulam. The percentage of cells in G1, S, and G2 phase are indicated. **C** Bar graph showing the percentage of cells in G1, S and G2 phase after 48 h of treatment with 3.3nM of PdB, 500nM of ThA and 3µM of Indisulam. Data represent the mean *±* SD of three independent experiments. **D** Representative micrographs of PANC-1 cells expressing tubulin-RFP and H2-GFP upon treatment with PdB for 24–72 h. Scale bar, 10 μm. **E** Bar graphs showing the percentage of aberrant and normal mitosis after 24–48 h of treatment with 3nM of PdB. Data represent the mean *±* SD of three independent experiments. **F** Bar graphs showing the percentage of mitotic index (mitotic cell with condensed chromosomes vs. total cells) after 24 h of treatment with PdB. Data represent the mean *±* SD of three independent experiments. **G** Frames extracted from time-lapse confocal analysis of control and PdB-treated PANC-1 cells. Scale bar, 10 μm. **H** Bar graphs show the duration of mitosis in cells treated or not with 3nM PdB. Statistical analyses were performed two-way ANOVA (**B**); one-way ANOVA (**E**); Student’s test (**F**, **H**) *P ≤ 0.05, **P ≤ 0.01, ***P ≤ 0.001

Functional annotation of the 623 common splicing-regulated genes indicated a strong enrichment in terms related to the G2/M checkpoint and mitotic spindle (Fig. 4A). Flow-cytometry analysis showed that treatment for 24–48 h with PdB or ThA, and to a lesser extent with indisulam, caused accumulation of PDAC cells in the G2/M phase of the cycle (Fig. 4B, C; Fig. S5D, E). Similar results were also observed with direct knockdown of either SF3B1 or RBM39 (Fig. S5F, G). To test whether execution of mitosis was impaired upon splicing inhibition, we engineered PANC-1 cells to stably co-express GFP-tagged histone H2b (GFP-H2) and RFP-tagged β-tubulin (RFP-TUB). Treatment with SF3B1 inhibitors caused extensive mitotic defects, with accumulation of condensed chromosomes onto monopolar spindles or of misaligned chromosomes onto bipolar spindles (Fig. 4D, E, Fig. S5H, I). Moreover, this phenotype was also observed in AsPC-1 cells (Fig. S5J, K), as well as by knocking down SF3B1 in PANC-1 (Fig. S6A, B).

Inhibition of SF3B1 lead to a significant increase in the mitotic index (Fig. 4F). Time-lapse analysis of cell cycle progression revealed that PdB-treated cells entered mitosis normally. However, due to aberrant spindle morphology, they were unable to properly complete chromosome segregation as compared to untreated cells (Fig. 4G, H). Furthermore, prolonged inhibition (Fig. S6C-F) or knockdown of SF3B1 (Fig. S6G, H) led to a strong accumulation of multinucleated cells.

**Fig. 5:**
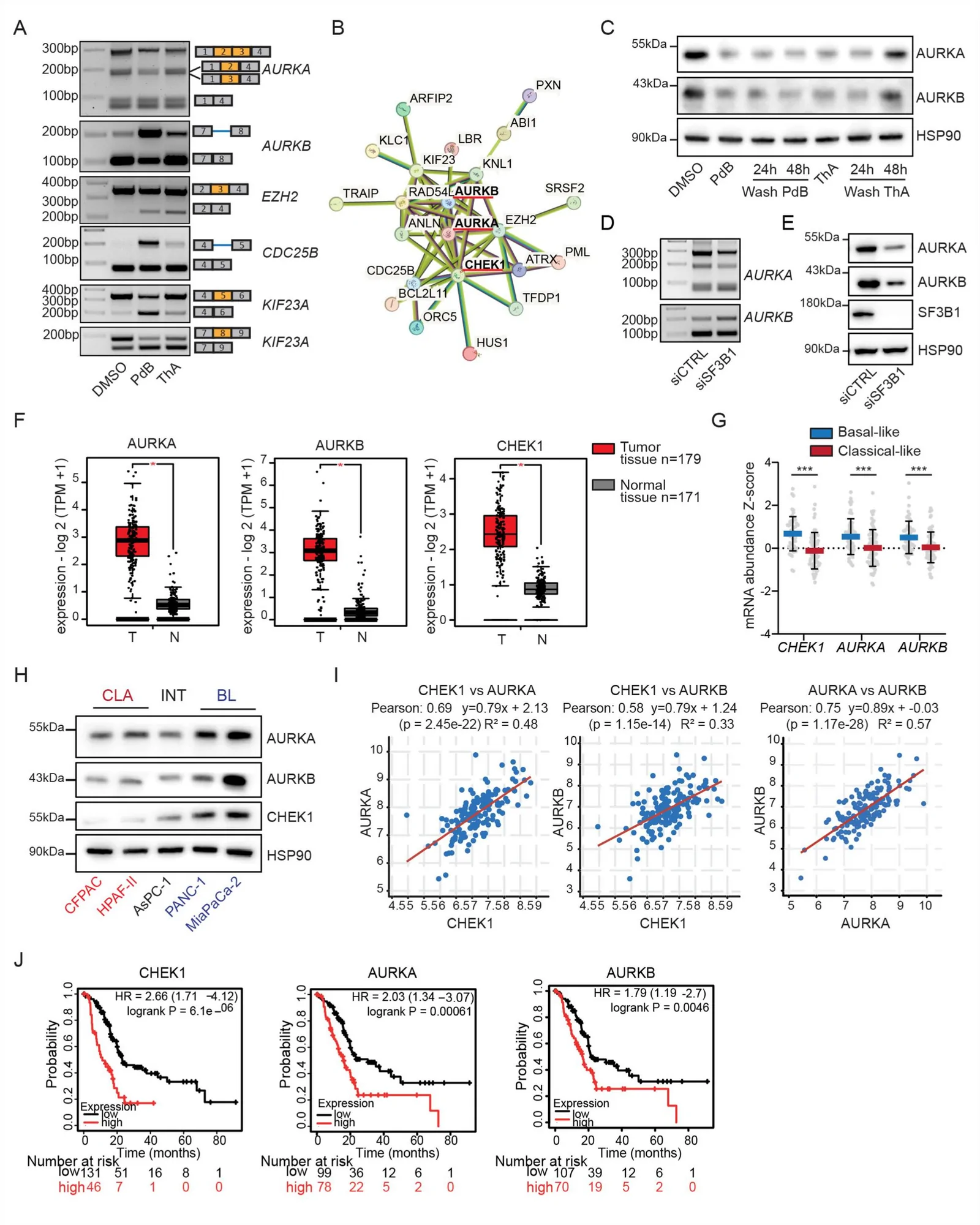
AURKA, AURKB and CHEK1 are associated with PDAC progression and poor prognosis. **A** RT-PCR analysis of the splicing events of representative mitotic genes affected by PdB (6 h) and ThA (8 h) in PANC-1 cells. **B** STRING analysis of the mitotic genes co-regulated by the three splicing inhibitors. **C** Representative Western Blot analysis for AURKA, AURKB and HSP90 protein levels in PANC-1 cells treated with PdB or ThA overnight followed by 24–48 h of drug removal. **D** RT-PCR analysis for representative splicing events affected by SF3B1 knockdown in PANC-1 cells. **E** Western Blot analysis for AURKA, AURKB, SF3B1 and HSP90 protein levels in PANC-1 cells transfected with siSF3B1 (48 h). **F** Box plots indicating the expression of AURKA, AURKB and CHEK1 genes in tumor and normal pancreatic tissues in The Cancer Genome Atlas (TCGA) dataset. **G** Graph showing the mRNA levels of CHEK1, AURKA and AURKB in Basal-like and Classical-like subtype in Clinical Proteomic Tumor Analysis Consortium (CPTAC) dataset. **H** Representative Western Blot analysis for AURKA, AURKB, CHEK1 and HSP90 protein levels in Classical-like (CL; CFPAC, HAPAF-II), intermediate (INT; AsPC-1) and Basal-like (BL; PANC-1, MiaPaCa-2) PDAC cell lines. **I** Correlation analysis of the expression of AURKA or AURKB with that of CHEK1 and between AURKA and AURKB in PDAC samples (TCGA, Firehose Legacy) using the cBioPortal database (https://www.cbioportal.org/). **J** Kaplan–Meier curve of overall survival for AURKA, AURKB and CHEK1 in PDAC patients from TGCA. Statistical analyses were performed Wilcoxon test and p. adjusted with the Benjamini-Hochberg (BH) adjustment (**F**), two-way ANOVA (**G**) *P ≤ 0.05, Pearson’s correlation coefficient (**I**); *P ≤ 0.05, **P ≤ 0.01, ***P ≤ 0.001

Coherent with the strong mitotic defects, treatment with SF3B1 inhibitors altered splicing of essential mitotic genes, including Aurora kinase A (*AURKA*) and B (*AURKB*), *CDC25B*, *KIF23* and *EZH2* (Fig. 5A). Search Tool for the Retrieval of Interacting Genes (STRING) analysis of the splicing-regulated “G2-M checkpoint” and “mitotic spindle” genes identified a core network of proteins centered on the mitotic kinases AURKA and AURKB and the DDR checkpoint protein kinase CHEK1 (Fig. 5B). As already observed for CHEK1 (Fig. 1F, Fig. S2B), transient inhibition of SF3B1 caused prolonged repression of AURKA and AURKB protein expression (Fig. 5C). Likewise, depletion of SF3B1 induced splicing alterations in these targets and decreased their protein abundance, providing evidence for a direct regulatory role of SF3B1 in their regulation (Fig. 5D, E). These findings imply that mitotic genes are highly sensitive to reduced splicing fidelity and that PDAC cells are dependent on efficient splicing for proper execution of mitosis.

High expression of genes involved in the G2/M cell cycle transition and mitotic spindle is a feature of a hybrid EMT state in PDAC cells, which is associated with more aggressive and metastatic phenotypes [45]. We found that expression of the *AURKA*, *AURKB* and *CHEK1*genes is significantly upregulated in PDAC with respect to normal pancreatic tissue (Fig. 5F). Moreover, their expression is higher in the more aggressive basal-like subtype with respect to classical PDAC samples (Fig. 5G). AURKA, AURKB and CHEK1 proteins are also expressed at higher level in basal-like cell lines (PANC-1 and MiaPaCa-2) with respect to classical (CFPAC-1 and HPAF-II) or intermediate (AsPC-1) cell lines (Fig. 5H). Furthermore, expression of these mitotic genes is highly correlated in PDAC samples (Fig. 5I), and it is significantly associated with worse prognosis in patients (Fig. 5J). Together, these observations suggest that concomitant upregulation of *CHEK1*, *AURKA* and *AURKB* expression contributes to PDAC malignancy.

### Combined inhibition of mitotic kinases recapitulates the cytotoxic effects of splicing inhibitors

**Fig. 6:**
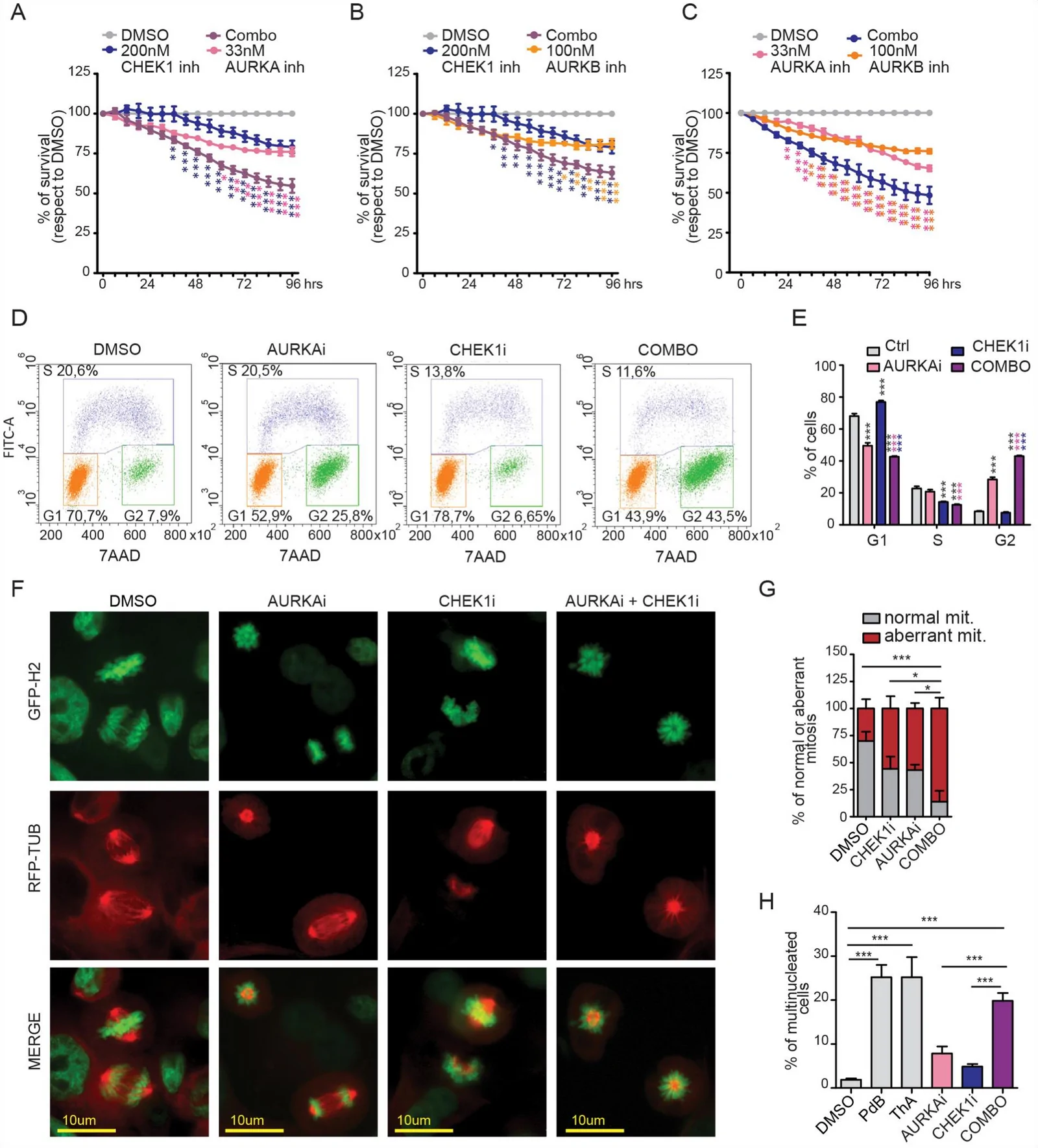
Combined targeting of AURKA and CHEK1 recapitulates the mitotic disruption caused by SF3B1 inhibitors. **A**-**C** Cell proliferation analysis of PANC-1 cells treated for 96 h with the indicated combinations of CHEK1i, AURKAi or AURKBi. Data represent the mean *±* SD of three independent experiments. Statistical analyses are reported in pink when referring to AURKAi treatment and in blue when referring to CHEK1i treatment (**A**), in orange when referring to AURKBi treatment and in blue when referring to CHEK1i treatment (**B**) or in orange when referring to AURKBi treatment and in pink when referring to AURKAi treatment (**C**). **D** FACS analysis showing DNA content (7AAD) and bromodeoxyuridine (BrdU) incorporation of PANC-1 cells treated for 72 h with 33nM of AURKAi, 200nM of CHEK1i and their combination. The percentage of cells in G1, S, and G2 phase are indicated. **E** Bar graph showing the percentage of cells in G1, S and G2 phase after treatment with the same doses and timing described in (**D**). Data represent the mean *±* SD of three independent experiments. **F** Representative micrographs of PANC-1 cells expressing tubulin-RFP and H2-GFP upon 24 h of treatment with AURKAi and CHEK1i alone or in combination. Scale bar, 10 μm. **G** Bar graphs showing the percentage of aberrant and normal mitoses after 24 h of treatment with AURKAi and CHEK1i alone or in combination. Data represent the mean *±* SD of three independent experiments. **H** Bar graphs showing the percentage of multinucleated cells after SF3B1 inhibitors (PdB and ThA) or AURKAi and CHEK1i alone and in combination. Data represent the mean *±* SD of three independent experiments. Statistical analyses were performed two-way ANOVA (**A**-**C**, **E**); one-way ANOVA (**G**, **H**) *P ≤ 0.05, **P ≤ 0.01, ***P ≤ 0.001

To test whether functional impairment of splicing-regulated mitotic kinases recapitulates the cytotoxic effects of splicing inhibitors, we employed clinically approved drugs targeting AURKA.

(Alisertib; AURKAi), AURKB (Barisertib; AURKBi) and CHEK1 (AZD7762; CHEK1i). When administered at suboptimal doses (Fig. S7A-C), combination of two of these inhibitors significantly increased the cytotoxic effects of each drug on PANC1 cells, with the strongest effects being observed for the AURKAi/CHEK1i and the AURKAi/AURKBi combinations (Fig. 6A-C). A significant increase in growth inhibition was also observed upon concomitant knockdown of either AURKA and CHEK1 or AURKB and CHEK1 (Fig. S7D-G).

Combined inhibition of AURKA and either CHEK1 or AURKB also increased the percentage of cells with a 4 N DNA content (Fig. 6D, E; Fig. S7H, I). Pharmacologic inhibition or knockdown of either AURKA or CHEK1 proteins in PDAC cells resulted in an increase in aberrant mitoses (Fig. 6F, G; Fig. S7J, K; Fig. S8A-D), albeit less pronounced than the effect of PdB (Fig. 4E). However, their combined administration recapitulated the strong mitotic defects caused by splicing inhibition (Fig. 6F, G; Fig. S7J, K) and the accumulation of multinucleated cells (Fig. 6H). Furthermore, the AURKAi/CHEK1i and AURKAi/AURKBi combination treatments induced increased DNA damage, as demonstrated by the accumulation of γH2AX foci (Fig. S9A-D). Importantly, gH2AX was present also in cells undergoing mitosis or stalled at aberrant mitoses, but not in mitoses of untreated cells nor in cells treated with single agents (Fig. S9E). These observations suggest that the combined treatment with a DDR (CHEK1i) and a mitotic (AURKAi) inhibitor leads to the accumulation of damaged mitotic cells and promotes mitotic catastrophe. In line with this notion, we observed a significant increase in apoptotic cells upon combined inhibition of AURKA and CHEK1 (Fig. S9F, G).

**Fig. 7:**
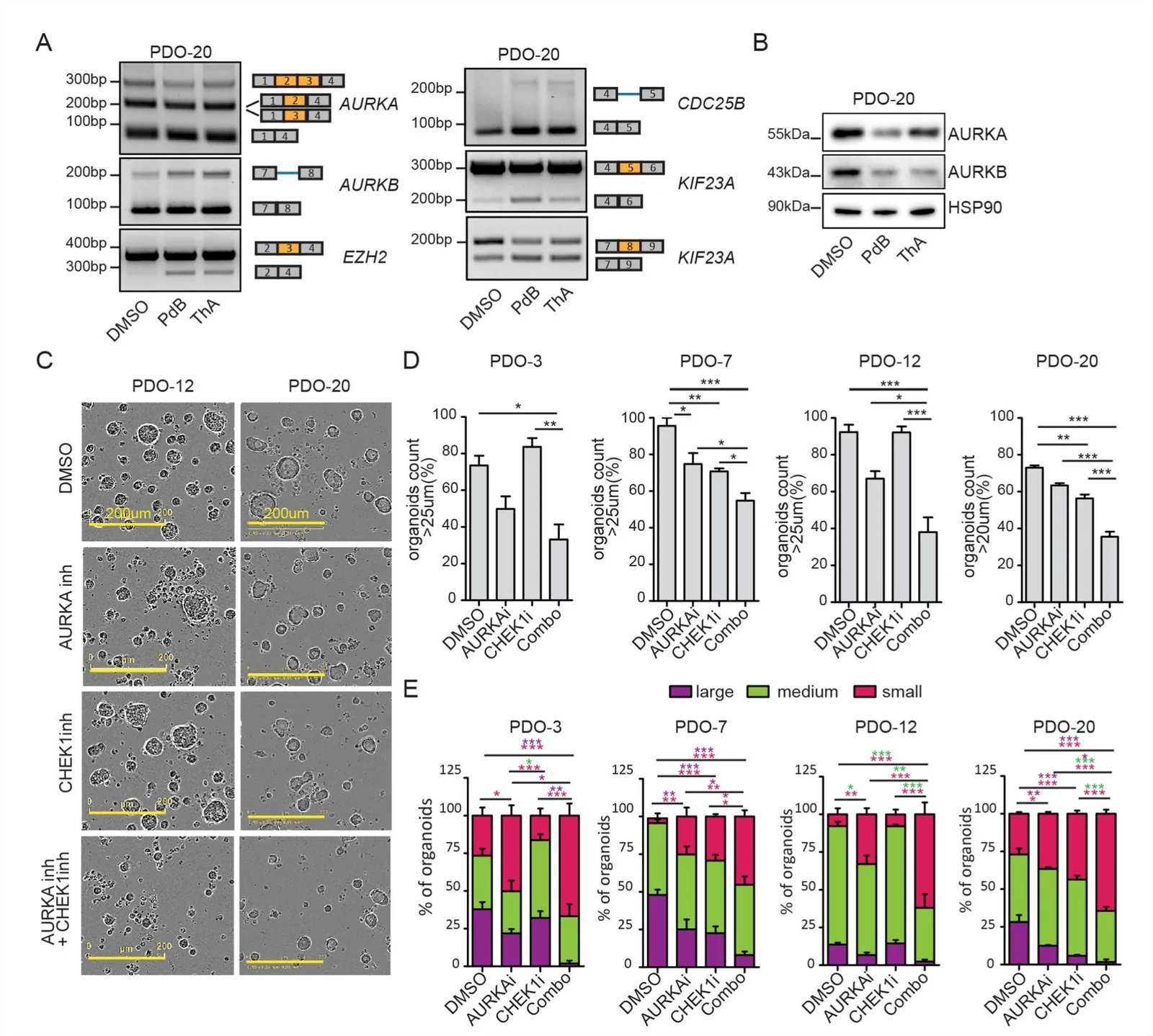
Combined inhibition of AURKA and CHEK1 enhance cytotoxicity in PDAC patient-derived organoids. **A** RT-PCR analysis for representative splicing events in mitotic genes affected by PdB and ThA (24 h) in PDO-20. **B** Representative Western Blot analysis for AURKA, AURKB and HSP90 protein levels in PDO-20 treated for 48 h with PdB and ThA. **C** Bright-field images of PDO cultures treated with 50nM of AURKAi (Alisertib), 25 nM (PDO-12) or 50 nM (PDO-20) CHEK1i either alone or in combination for 5 days. Scale bar, 200 μm. **D-E** Percentages of organoids that have a size greater than 25 μm (PDOs-3-7-20) or 20 μm (PDO-20) (**D**) and percentage of large, medium, and small PDOs treated with 50nM AURKAi (PDO-3-7-12-20), 50nM (PDO- 3-7-20) or 25nM (PDO-12) CHEK1i either alone or in combination (combo) (**E**). Statistical analyses are reported in purple, green and pink when referring to large, medium and small organoids respectively. Statistical analyses were performed one-way ANOVA (**D**); two-way ANOVA (**E**); *P ≤ 0.05, **P ≤ 0.01, ***P ≤ 0.001

SF3B1 inhibitors caused the same alterations in splicing and expression of mitotic genes also in PDAC PDOs with different genetic background (Fig. 7A, B; Fig. S10A, B), indicating their reproducible and general effect on this pathway. Combined treatments with suboptimal doses of AURKAi and CHEK1i synergically reduced the number and size of PDOs with respect to the effect of each drug alone, with the exception of PDO-7 were we found an additive effect (Fig. 7C-E; Fig. S10C, H). Similarly, combination of CHEK1i with AURKBi elicited either synergic (PDO-3 and PDO-12) or additive (PDO-7 and PDO-20) effects (Fig. S10D-G). Together, these data suggest that splicing inhibitors strongly impair cell proliferation by simultaneously disrupting the proper expression and function of multiple DDR and mitotic proteins in PDAC cells.

## Discussion

Splicing is highly deregulated in cancer cells and oncogenic splice variants affect all their hallmarks [3]. Thus, counteracting such oncogenic splicing events represents a potentially valuable therapeutic strategy. RNA-based therapies that correct splicing defects have now entered the clinic for neuromuscular and neurodegenerative diseases [46]. However, since these diseases rely on a single splicing defect, the therapeutic target was more clearly defined. In the case of cancer, where multiple splicing defects generally occur concomitantly, successful splicing-targeting therapies have not yet been established. Nevertheless, due to the frequent mutation or aberrant expression of genes encoding core components of the splicing machinery, the spliceosome itself is being evaluated as potential therapeutic target for some cancer types [13–15]. In this regard, the U2 snRNP, which recognizes the 3’ splice site, has emerged as a targetable node in splicing regulation [13–15] and was also identified as a regulator of genomic stability and replication stress response in cancer cells [26, 27]. Interestingly, a recent report demonstrated that mutant KRAS relies on the upregulation of splicing factors, including SF3B1 and RBM39, for its oncogenic activity, and that KRAS-mutated cancers are highly sensitive to SF3B1 inhibition [47]. Furthermore, SF3B1 inhibitors were particularly efficacious in PDAC mouse models harboring both KRAS^G12D^ and TP53^R175H^, two of the most common mutations found in patients [4]. Herein, we found that many genes encoding for U2 snRNP proteins are upregulated in advanced, metastatic PDAC. Furthermore, our work indicates that PDAC cells are highly sensitive to low doses of U2 snRNP-targeting drugs or to SF3B1 and RBM39 knockdown. Splicing inhibition exerts its cytotoxic effects by disrupting the DDR and the mitotic spindle checkpoint, thus leading to impairment of mitosis and onset of apoptotic cell death. Importantly, PDOs with a heterogeneous mutational spectrum were also highly sensitive to these drugs, suggesting the possible translational relevance of our findings. Thus, our data point to U2 snRNP as a potentially valuable target for PDAC. Moreover, it also identifies splicing targets whose combined inhibition may represent an actionable vulnerability for PDAC, as clinically approved drugs for these proteins already exist.

The enhanced sensitivity of the DDR and of the mitotic spindle checkpoint to perturbation of the splicing fidelity is likely associated with the structure of the genes involved in these processes. Indeed, the large overall size coupled with a smaller exon and intron size leads to the presence of a higher number of exon-intron junctions in splicing-sensitive genes. Moreover, they genes are highly expressed in PDAC cells. Thus, the continuous generation of many transcripts that require multiple splicing events to be fully processed possibly imposes a stricter dependency on high fidelity of 3’ splice site recognition and efficient splicing. We also found that ECs, which are mainly skipped under treatment with all splicing inhibitors, are charactered by weak 5’ splice sites and a low GC content, which is typical of weak exons [48]. Noteworthy, the weak 5’ splice site may represent an actionable vulnerability in the presence of drugs that decrease the fidelity of 3’ splice sites. Indeed, while reducing the 5’ splice site strength of a PdB-insensitive exon did not affect its splicing under basal conditions, it conferred dose-dependent sensitivity to splicing inhibition. The introns that are susceptible to retention upon splicing perturbation were also significantly smaller and harbored weak 5’ splice sites. However, in this case they displayed a high GC content which, together with their small size, may confer them an exon-like appearance.

Our data suggest the existence of a link between splicing regulation, DNA repair and control of mitotic progression. We hypothesize that this link allows cells to synchronize DNA replication, when most endogenous DNA damage events occur, and mitosis with efficient processing of transcripts. Thus, given its essential role in cell fitness, splicing fidelity could represent a new form of cell cycle checkpoint. In this scenario, when perturbation of splicing occurs, cells would rapidly become unable to produce proteins involved in DNA repair and to recognize misalignment of chromosomes, thus triggering programmed cell death in mitosis and avoiding aberrant cell divisions.

Splicing inhibitors were shown to synergize with chemotherapy, targeted therapies and immunotherapies in other cancer types [22–27, 49]. Herein, we found that SF3B1 inhibitors cause a prolonged repression of DDR and mitotic proteins, even after their removal, when most splicing defects are rescued. This evidence indicates that DDR and mitotic genes display an enhanced and persistent vulnerability to transient splicing inhibition. We tested whether this feature could be exploited to increase the cytotoxic effects of drugs in use or in trials in the clinic. For instance, the prolonged repression of crucial DDR proteins, like BRCA1/2 and CHEK1, suggested to us that a transient splicing perturbation could generate a BRCAness phenotype in PDAC cells that are otherwise wild type for *BRCA1* and *BRCA2*. Indeed, PdB and ThA significantly enhanced the response of HRP PDAC cells and organoids to talazoparib. Moreover, the same effect was observed by targeting CHEK1 activity, one of the DDR proteins that is strongly repressed by SF3B1 inhibition. These data suggest that either global or selective - antisense oligonucleotide-guided - inhibition of splicing could be useful to expand the cohort of PDAC patients that benefit of PARPi, which is now limited to those harboring *BRCA1/2* or *PALB2* mutations [43, 50].

We also found that SF3B1 inhibitors strongly impaired the execution of mitosis, with aberrant assembly of the spindle and misalignment of chromosomes at metaphase. The mitotic defects are likely related to the direct effect on the expression of key mitotic genes, such as *AURKA* and *AURKB*. SF3B1 inhibition or knockdown causes the retention of intron 7 in *AURKB*, which likely accounts for the reduced expression of AURKB protein. On the other hand, the exons 2 and 3 of *AURKA* encode for the 5’ untranslated region of the transcript. Inclusion of these exons is upregulated in cancer cells and promote translation of the AURKA protein [51, 52]. Thus, their repression upon U2 snRNP perturbation likely underlies the reduced expression of this protein kinase. Importantly, a small interfering RNA (siRNA) targeting *AURKA* exon 2 displayed strong anticancer activity in vitro and in vivo [52], indicating the translational relevance of this splicing event. We found that pharmacologic inhibition of single mitotic kinases was not sufficient to efficiently block PDAC cell proliferation. Nevertheless, combination of two of these drugs, and particularly the combination of AURKAi with either CHEK1i or AURKBi, exerted much stronger effects and closely recapitulated the mitotic defects and apoptotic death caused by U2 snRNP inhibitors.

## Conclusions

Our study suggests that the concomitant and persistent repression of multiple DDR and mitotic proteins by treatment with U2 snRNP inhibitors causes an unsurmountable mitotic block, likely due to the profound alteration of the mitotic spindle dynamics observed in our immunofluorescence studies. These findings point to the U2 snRNP as a deregulated node and actionable target in PDAC and identify key features of DDR and mitotic genes that render them particularly susceptible to perturbation of splicing fidelity. Nevertheless, the translational relevance of our findings will require confirmation using in vivo models of PDAC, to assess tolerability of the treatments and maintenance of the in vitro effects. In this regard, recent promising data reported that splicing regulation is an actionable vulnerability in mouse models of oncogenic KRAS-driven cancers [47]. Furthermore, administration of SF3B1 inhibitors was tolerated in PDAC mouse models and significantly improved survival [4]. Likewise, indisulam was shown to specifically potentiate the activity of PARPi in mouse models of ovarian cancer, without overt non-specific toxicity [24]. Tolerability and efficacy in mouse PDAC models were also assessed for the AURKAi [53] and the CHEK1i, which also enhanced the cytotoxic activity of gemcitabine [54]. However, it remains to be established whether splicing inhibition in PDAC in vivo models is also able to enhance the effect of PARPi or of mitotic inhibitors, as suggested by our study.

## Supplementary Information

Supplementary Material 1: Supplementary Information include Additional Files 1-3.

Supplementary Material 2.

Supplementary Material 3.

## Data Availability

The datasets generated during the current study have been deposited in the GEO (Gene Expression Omnibus) under the accession number GSE312205. Single cell sequencing data from previously published dataset [37] were downloaded from the GEO dataset with the accession number (GSE205013).

## Abbreviations

7-AAD: 7-aminoactinomycin D
BrdU: Bromodeoxyuridine
CPTAC: Clinical Proteomic Tumor Analysis Consortium
DDR: DNA damage response
GFP-H2: GFP-tagged H2b histone
GO: Gene ontology
HRP: HR proficient
MAPK: Mitogen activated protein kinase
PARPi: Poly-ADP-ribose polymerase inhibitors
PDAC: Pancreatic ductal adenocarcinoma
PdB: Pladienolide B
PDOs: Patient-derived organoids
pre-mRNAs: Precursor messenger RNAs
QKI: Quaking
RBP: RNA binding protein
RFP-TUB: RFP-tagged b-tubulin
SF3B: Splicing factor 3 B
siRNA: Small interfering RNA
ThA: Thailanstatin A
U2 snRNP: U2 small nuclear ribonucleoprotein
