# Supplementary material for: U2 snRNP inhibition hampers pancreatic cancer cell viability by disrupting DNA repair proficiency and mitotic checkpoint integrity
Source: J Exp Clin Cancer Res. 2026 Jul 31;45:216. doi: 10.1186/s13046-026-03795-3 (PMC13660249; doi:10.1186/s13046-026-03795-3)
Supplement: Supplementary file 1 [file 13046_2026_3795_MOESM3_ESM.docx]

**Additional file 3**

**This file includes 10 Supplementary Figures and their Figure legends.**

**Supplementary Figure 1. A)** Dot plot showing upregulated (red) and downregulated (blue) of the indicated U2 snRN, U2AF complex and SF1 expression level in primary and metastatic tumor by single-cell transcriptomic analysis.  **B)** Violin plot of SF3A3, SF3B2 and SF1 expression level in metastatic and not cells identified by single-cell transcriptomic analysis. **C)** Violin plot of SF3A3, SF3B2 and SF1 expression level in tumors classified as classical, intermediate, and basal-like by single-cell transcriptomic analysis. Statistical analyses were performed by Wilcoxon test and p.adjusted with the Benjamini-Hochberg (BH) adjustment (B-C). **D)** Kaplan–Meier curve of disease-free survival for U2 snRNP signature of genes upregulated in metastatic PDAC patients (*SF3A1*, *SF3A2*, *SF3A3*, *SF3B2*, *SF3B3*, *SF3B4*, *SF3B6* and *PHF5A*) from TGCA **E)** Dose-response curves illustrating the growth inhibitory effects of the splicing targeting drugs PdB, ThA and Ind on PANC-1, MiaPaCa-2 and AsPC-1 cells. Cell growth was evaluated as measure of cells after 96h of treatment with respect to vehicle (DMSO) treated cells, set to 100%. Half-maximal inhibitory concentration (IC50) values were determined from fitting curves using GraphPad Prism. Data represent the mean ± SD of three independent experiments. **F)** Representative Western Blot analysis for PARP-1, Cleaved caspase 3 and HSP90 protein levels in PANC-1, MiaPaCa-2 and AsPC-1 cells treated for 72h with PdB, ThA, and Ind. **G)** RT-PCR analysis for representative splicing events affected by PdB, ThA and Ind in MiaPaCa-2 and AsPC-1 cells. **H)** Representative Western Blot analysis for BRCA1, BRCA2, CHEK1 and HSP90 protein levels in MiaPaCa-2 and AsPC-1 cells treated with PdB (O.N), ThA (O.N) and Ind (24 h). **I)** Representative Western Blot analysis for γH2AX and HSP90 protein levels in MiaPaCa-2 and AsPC-1 cells treated for 72h with splicing inhibitors.

**Supplementary Figure 2.** **A-B)** RT-PCR analysis for *BRCA1* and *CHEK1* splicing events (A) and representative Western Blot analysis for BRCA1 and CHEK1 protein levels (B) in PANC-1 cells treated with 3.3nM PdB for O.N (T0) and 24h-48h after treatment removal. **C)** Bright-field images of PANC-1 cells pre-treated for 24h with suboptimal doses of splicing inhibitors (PdB 1 nM; ThA 100 nM; Ind 1 uM) or treated with Talazoparib (Tal) (200 nM) either alone or in combination. Scale bar, 300µm **D)** Cell proliferation analysis of PANC-1 cells pre-treated with splicing inhibitors the same doses and timing described in C. Cell growth was evaluated as measure of cells after 96h of treatment with respect to vehicle (DMSO) treated cells, set to 100%. Schematic representation of the experimental workflow is shown in the figure. Data represent the mean ± SD of three independent experiments. Statistical analyses are reported in green when referring to pre-treated for 24h with suboptimal doses of splicing inhibitors and in blue when referring to Tal treatment. **E)** Cell proliferation analysis of PANC-1 cells treated for 24h with splicing inhibitors (PdB 1 nM; Th.A 100 nM; Ind 1 uM) and then with a dilution 1:10 of these drugs (PdB 0,1 nM; Th.A 10 nM; Ind 100 nM) together at 200nM of Tal. Schematic representation of the experimental workflow is shown in the figure. Data represent the mean ± SD of three independent experiments. Statistical analyses are reported in green when referring to splicing inhibitors treatment and in blue when referring to Tal treatment. Statistical analyses were performed by two-way ANOVA (D-E) *P <0.05; **P <0.01; ***P<0.001.

**Supplementary Figure 3. A)** Representative bright-field images of PDOs cell lines from biopsies of primary PDAC. Scale bar, 20µm. **B)** H&E staining and immunohistochemistry staining of KRT-18, MUC-13 and Ki‐67 on PDO 3-7-12-20. Scale bar, 20µm. **C)** RT-PCR analysis for representative splicing events affected by PdB, ThA and Ind (24h) in PDO 7- 12-20. **D)** Representative Western Blot analysis for BRCA1, BRCA2, CHEK1 and HSP90 protein levels in PDO-7-12-20 treated for 48h with splicing inhibitors.

**Supplementary Figure 4**. **A)** Bright-field images of PDO-3-20 cultures treated with 200nM of Talazoparib (Tal) and 1nM PdB alone or in combination (combo) for 5 days. Scale bar, 200 μm. **B-C)** Percentages of organoids that have a size greater than 30µm (PDO-3), 25µm (PDO-20) B) and percentage of large, medium, and small PDO-3-20 (C) for conditions described in (A). Statistical analyses are reported in purple, green and pink when referring to large, medium and small organoids respectively. Synergism between drugs was evaluated by the Bliss independence model, setting the Δ between expected and observed >0.1 as threshold. **D)** Percentages of organoids that have a size greater than 20µm (PDO-3-7-12-20), 25µm (PDO-7) after treatment with 200nM Tal and CHEK1i (AZD7762) alone or in combination (combo) for 5 days. Synergism between drugs was evaluated by the Bliss independence model, setting the Δ between expected and observed >0.1 as threshold. **E)** Percentage of large, medium, and small PDO-3-7-12-20 for conditions described in (D). Statistical analyses are reported in purple, green and pink when referring to large, medium and small organoids respectively. Statistical analyses were performed by one-way ANOVA (B-D) two-way ANOVA (C-E) *P <0.05; **P <0.01; ***P<0.001.

**Supplementary Figure 5. A)** Box plots representing comparison of GC content between 623 common splicing inhibitor (genes SI) regulated genes, 2987 genes regulated only by one of the splicing targeting drugs (genes other SI) and 9062 genes not regulated by any of them. **B)** Box plots representing GC content in previous and following EC regulated between 228 common splicing inhibitor (EC SI) regulated EC, 4445 EC regulated only by one of the splicing targeting drugs (EC other SI) and 122043 EC not regulated by any of them. **C)** Box plots representing Brach point score in the categories described in B. **D-E)** FACS analysis showing DNA content (7AAD) and bromodeoxyuridine (BrdU) incorporation (D) and bar graph shows the percentage of cells in G1, S and G2 phase (E) in PANC-1 cells treated for 24h with 3.3nM of PdB, 500nM of ThA and 3.3uM of Ind. Data represent the mean ± SD of three independent experiments. **F-G)** FACS analysis showing 7AAD and BrdU incorporation (D) and bar graph shows the percentage of cells in G1, S and G2 phase (E) in PANC-1 cells transfected with the corresponding siRNAs (48). Data represent the mean ± SD of three independent experiments. **H)** Representative micrographs of PANC-1 cells tagged with tubulin in RFP and H2 with GFP upon 24/48h of treatment with ThA. Scale bar, 10 μm. **I)** Bar graphs show the percentage of aberrant or normal mitosis after 24/48h of treatment with ThA in PANC-1 cells. Data represent the mean ± SD of three independent experiments. **J)** Representative micrographs of AsPC-1 cells tagged with tubulin in RFP and labelled with Hoechst upon 24h of treatment with 3.3nM PdB and 500nM ThA. Scale bar, 10 μm. **K)** Bar graphs show the percentage of aberrant or normal mitosis after 24h of treatment with PdB and ThA in AsPC-1 cells. Data represent the mean ± SD of three independent experiments. Statistical analyses were performed by Wilcoxon test and p.adjusted with the Benjamini-Hochberg (BH) adjustment (A-C); two-way ANOVA (E,G, I, K); *P <0.05; **P <0.01; ***P<0.001.

**Supplementary Figure 6. A)** Representative micrographs of PANC-1 cells tagged with tubulin in RFP and H2 with GFP transfected with siRNA for SF3B1 and CTRL (48h). Scale bar, 10 μm. **B)** Bar graphs show the percentage of aberrant or normal mitosis in PANC-1 cells transfected with the corresponding siRNAs. Data represent the mean ± SD of three independent experiments. **C)** Representative micrographs of PANC-1 cells tagged with tubulin in RFP and H2 with GFP upon 72h of treatment with PdB and ThA. Scale bar, 10 μm. **D)** Representative micrographs of AsPC-1 cells tagged with tubulin in RFP and labelled with Hoechst upon 72h of treatment with PdB and ThA. Scale bar, 10 μm**. E-F)** Bar graph show the percentage of multinucleated cells after 72h of treatment with PdB and ThA in PANC-1 (C) and in AsPC-1 (D) cells. Scale bar, 10 μm. Data represent the mean ± SD of three independent experiments. **G)** Representative micrographs of PANC-1 cells tagged with tubulin in RFP and H2 with GFP transfected with siRNA for SF3B1 and CTRL (72h). Scale bar, 10 μm. **H)** Bar graphs show the percentage of multinucleated PANC-1 cells transfected with the corresponding siRNAs. Data represent the mean ± SD of three independent experiments. Statistical analyses were performed by one-way ANOVA (D, F, H); two-way ANOVA (B) *P <0.05; **P <0.01; ***P<0.001.

**Supplementary Figure 7. A-C)** Cell proliferation analysis of PANC-1 cells treated with dosage of AURKAi (Alisertib) (A), AURKBi (Barisertib) (B) and CHEK1i (AZD7762) (C). Cell growth was evaluated as measure of cells after 72h of treatment with respect to vehicle (DMSO) treated cells, set to 100%. **D, F**) Representative Western Blot analysis for CHEK1, AURKA, AURKB and HSP90 protein levels in PANC-1 cells transfected with the indicated siRNAs (48h) **E, G**) Cell proliferation analysis of PANC-1 cells transfected with the indicated siRNAs. Data represent the mean ± SD of three independent experiments. Statistical analyses are reported in pink when referring to AURKA knockout and in blue when referring to CHEK1 knockout (E), in orange when referring to AURKB knockout and in blue when referring to CHEK1 knockout G). **H-I)** FACS analysis showing DNA content (7AAD) and bromodeoxyuridine (BrdU) incorporation (D) and bar graph shows the percentage of cells in G1, S and G2 phase (E) in PANC-1 cells treated for 24h with 33nM of AURKAI, 100nM of AURKBi alone and in combination. Data represent the mean ± SD of three independent experiments. **J)** Representative micrographs of AsPC-1 cells tagged with tubulin in RFP and labelled with Hoechst upon 24h of treatment with AURKAi and CHEK1i alone or in combination. Scale bar, 20 μm. **K)** Bar graphs show the percentage of aberrant or normal mitosis after 24h of treatment with AURKAi and CHEK1i in AsPC-1 cells. Data represent the mean ± SD of three independent experiments. Statistical analyses were performed by one-way ANOVA (I) and two-way ANOVA (E, G, K) *P <0.05; **P <0.01; ***P<0.001.

**Supplementary Figure 8. A)** Representative micrographs of PANC-1 cells tagged with tubulin in RFP and H2 with GFP transfected with siRNA for *AURKA, AURKB, CHEK1* and CTRL (48h). Scale bar, 10 μm. **B)** Bar graphs show the percentage of aberrant or normal mitosis in PANC-1 cells transfected with the corresponding siRNAs. Data represent the mean ± SD of three independent experiments. **C)** Representative micrographs of PANC-1 cells tagged with tubulin in RFP and H2 with GFP transfected with SF3B1 and non-targeting (CTRL) siRNAs (72h). Scale bar, 10 μm. **D)** Bar graphs show the percentage of multinucleated PANC-1 cells transfected with the indicated siRNAs. Data represent the mean ± SD of three independent experiments. Statistical analyses were performed by one-way ANOVA (D); two-way ANOVA (B) *P <0.05; **P <0.01; ***P<0.001.

**Supplementary Figure 9.** **A-D)** Representative micrographs of immunofluorescence assay for γH2AX in PANC-1 cells transfected with the indicated siRNAs (48h) (A, C) and the relative quantification of γH2AX positive cells (B, D). Scale bar, 10 μm. Data represent the mean ± SD of three independent experiments. Statistical analyses were performed by one-way ANOVA (B, D); *P <0.05; **P <0.01; ***P<0.001.**E**) Representative micrographs of γH2AX immunofluorescence analysis in PANC-1 cells transfected with the indicated siRNAs (24h). Arrows in the merge panels point to mitotic cells. Scale bar, 10 μm. **F-G)** Representative micrographs of cleaved caspase-3 (F) in PANC-1 cells treated for 72h with AURKAi and CHEK1i alone or in combination and relative quantification of positive cells (G). Scale bar, 50μm. Statistical analyses were performed by one-way ANOVA (G).

**Supplementary Figure 10. A)** RT-PCR analysis for representative splicing events affected by PdB and ThA (24h) in PDO-3-7-12. **B)** Representative Western Blot analysis for AURKA, AURKB and HSP90 protein levels in PDO-3-7-12 treated for 48h with PdB and ThA. **C)** Bright-field images of PDO cultures treated with 50nM of AURKAi (Alisertib), 50nM (PDO-3-7) CHEK1i alone or in combination for 5 days. Scale bar, 200 μm for PDO3 and 400µm for PDO-7. **D)** Bright-field images of PDO-3-7-12-20 cultures treated with 50nM (PDO 3-7-12) / 100nM (PDO-20) of AURKBi (Barisertib), and 50nM (PDO-3-20) / 25nM (PDO 7-12) of CHEK1i alone or in combination for 5 days. Scale bar, 200 μm (PDO-3-12-20) and 400µm for PDO-7. **E-F)** Percentages of organoids that have a size greater than 25µm (PDOs-3-7-20) or 20µm (PDO-20) (E) and percentage of large, medium, and small PDOs (F) for conditions described in (D). Statistical analyses are reported in purple, green and pink when referring to large, medium and small organoids respectively. Statistical analyses were performed one‐way ANOVA (E); two-way ANOVA (F); *P ≤ 0.05, **P ≤ 0.01, ***P ≤ 0.001. **G-H)** Synergism between drugs was evaluated by the Bliss independence model, setting the Δ between expected and observed >0.1 as threshold. Values between 0 and 0.1 indicate additivity.
